# Experimental and Clinical Researches Applied to Physiology and Pathology

**Published:** 1857-01

**Authors:** Ed. Brown-Sequard

**Affiliations:** Laureate of the Académie des Sciences of France, Vice President of the Société de Biologie, ex-Secretary of the Société Philomathique of Paris, ex-Professor of the Institutes of Medicine in the Richmond Medical College, &c.


[Communicated for the Boston Medical and Surgical Journal.]

*Experimental and Clinical Researches Applied to Physiology and Pathology.* By ED. BROWN-SEQUARD, M. D., Laureate of the *Académie des Sciences* of France, Vice President of the *Société de Biologie*, ex-Secretary of the *Société Philomathique* of Paris, ex-Professor of the Institutes of Medicine in the Richmond Medical College, &c.

From August, 1852, to August, 1853, I published in the *Medical Examiner*, of Philadelphia, a series of thirty-three short papers, which was afterwards connected in one volume, under the title : "*Experimental Researches Applied to Physiology and Pathology.*" The following article is the first of a second series of papers, which, with the preceding series which has appeared in Philadelphia, will form a complete summary of all my original researches in various branches of the medical sciences.

*I. Artificial Production of an Epileptiform Affection in Animals, and Etiology and Treatment of certain forms of Epilepsy in Man.*

Six years ago, I discovered that certain alterations of the spinal cord, upon mammals, produce, after a few weeks, a convulsive affection, resembling epilepsy. (See *Comptes Rendus de la Soc. de Biol.*, 1. ii., pp. 105 and 169—1850.) Since that time, I have found many new facts concerning this affection; and lately, in comparing the results of my experiments with what has been observed in man, in many cases of epilepsy, I have been led to some conclusions, which are I think, very important, as regards the etiology, the nature and the treatment of epilepsy. Although some of the results of my experiments have already been published (see my *Exper. Researches applied to Physiology and Pathology*, pp. 36 and 80, the *Archives de Médec., etc.*, Fevrier, 1856; and the *Moniteur des Hopitaux*, Oct., 1856, p. 954), I will relate them here, as I shall have to make use of them when I expose my views upon the pathology and treatment of epilepsy. I will also give a detailed account of some of the facts I have observed in animals, because these facts throw a great deal of light upon the phenomena of epilepsy in man.

§ I. I have found that the following kinds of injury to the spinal cord are able to produce epilepsy, or at least a disease resembling epilepsy, in animals belonging to different species, but mostly upon guinea-pigs.

1st. A complete transversal section of a lateral half of this organ.

2d. A transversal section of its two posterior columns, of its posterior cornua of gray matter, and of a part of the lateral columns.

3d. A transversal section of either the posterior columns or the lateral, or the anterior alone.

4th. A complete transversal section of the whole organ.

5th. A simple puncture.

Of all these injuries, the first, the second and the fourth seem to have more power to produce epilepsy than the others. The first particularly, *i. e.*, the section of a lateral half of the spinal cord, seems to produce constantly this disease in animals that live longer than three or four weeks after the operation. After a section of either the lateral, the anterior or posterior columns alone, epilepsy rarely appears, and it seems that in the cases where it has been produced, there has been a deeper incision than usual, and that part of the gray matter has been attained. In other experiments, few in number, the section of the central gray matter (the white being hardly injured) has been followed by this convulsive disease. I have seen it but very rarely after a simple puncture of the cord.

It is particularly after injuries to the part of the spinal cord which extends from the seventh or eighth dorsal vertebra to the third lumbar, that epilepsy appears.

§ II. Usually this affection begins during the third or fourth week after the injury. In some cases I have seen it beginning during the second week, and even one or two days before. At first the fit consists only in a spasm of the muscles of the face and neck, either on one or the two sides, according to the transversal extent of the injury. One eye or both are forcibly shut, the head is drawn towards one of the shoulders, and the mouth opened by the spasm of some of the muscles of the neck. This spasmodic attack quickly disappears.

After a few days the fit is more complete, and all parts of the body, which are not paralyzed, have convulsions. According to the seat of the injury, the parts that have convulsions greatly vary. When the lesion is near the last dorsal vertebræ or the first lumbar, and consisting of a section of a lateral half of the spinal cord, convulsions take place everywhere, except only the posterior limb on the side of the injury. If the lesion consists of the section of the two posterior columns and a part of the lateral columns, and of the gray matter, convulsions take place everywhere without exception, but with much more violence in the anterior parts of the body. When the lesion exists at the level of the last dorsal vertebræ and consists in a transversal section of the two anterior or of the two lateral columns, convulsions are ordinarily limited to the anterior parts of the body; but it is a very interesting fact that they are not always confined to these parts, the two posterior limbs having sometimes very strong tetanic spasms, at the same that there are clonic convulsions in the anterior limbs. After a transversal section of the central gray matter, or of the whole spinal cord, in the dorsal region, convulsions are limited to either the anterior or the posterior parts of the body.

§ III. Convulsions may come either spontaneously, or after certain excitations. The most interesting facts concerning these fits is that it is possible, and even very easy, to produce them by two modes of irritation. If we take two guinea pigs, one not having been submitted to any injury of the spinal cord, and the other having had this organ injured, we find, in preventing them from breathing for two minutes, that convulsions come in both; but if we allow them to breathe again, the first one recovers almost at once, while the second continues to have violent convulsions for two or three minutes and sometimes more. There is another mode of giving fits to the animals which have had an injury to the spinal cord. Pinching of the skin in certain parts of the face and neck is always followed by a fit. If the injury to the spinal cord consists only in a transversal section of a lateral half, the side of the face and neck which, when irritated, may produce the fit, is on the side of the injury; *i. e.*, if the lesion is on the right side of the cord, it is the right side of the face and neck which are able to cause convulsions, and *vice versa*. If the two sides of the cord have been injured, the two sides of the face and neck have the faculty of producing fits, when they are irritated. No other part of the body but a portion of the face and neck has this faculty. In the face, the parts of the skin animated by the opthalmic nerve cannot cause the fits; and of the two other branches of the trigeminal nerve, only a few filaments have the property of producing convulsions. Among these filaments, the most powerful, in this respect, seem to be some of those of the suborbitary and of the auriculo-temporalis. A few filaments of the second, and perhaps of the third cervical nerve, have also this property of producing fits. In the face, the following parts may be irritated without inducing a fit: the nostrils, the lips, the ears, and the skin of the forehead and that of the head. In the neck, there is the same negative result when an irritation is brought upon the parts in the neighborhood of the median line, either in front or behind. On the contrary, a fit always follows an irritation of some violence when it is made in any part of a zone limited by the four following lines: one uniting the ear to the eye; a second from the eye to the middle of the length of the inferior maxillary bone; a third which unites the inferior extremity of the second line to the angle of the inferior jaw; and a fourth which forms half a circle, and goes from this angle to the ear, and the convexity of which approaches the shoulder.

§ IV. Can we attribute to the great degree of sensibility of the face and of the neck the property exclusively possessed by these parts to produce fits in animals which have had their spinal cord injured? In other words, is it in consequence of the pain felt, that there are fits in these circumstances? This explanation is quite in opposition with the following facts: 1st. When the injury exists only in one of the lateral halves of the cord, the face and neck on the other side have not the power of producing fits, whatever is the degree of the irritation upon them. 2d. In the same case, the posterior limb on the side where the cord is injured, is in a state of hyperæsthesia, and, nevertheless, the most violent irritations upon this limb do not produce fits. 3d. It is sometimes sufficient to touch the face or the neck, or even to blow upon them, to produce the fits. Therefore, unless we admit that there is an extraordinary degree of hyperæsthesia in the parts which possess the faculty of producing the convulsions when they are irritated, we must admit that it is not the pain which causes these convulsions. There does not seem to be more sensibility in these parts than in other parts of the body. When a fit, or rather a series of fits, have taken place, and when, consequently, the power of having them is much diminished, it is easy to ascertain that these parts seem not to be more sensitive than others. The animal does not cry more when they are pinched or galvanized, than when other parts are irritated in the same way.

The production of fits by the irritation of certain parts of the neck and face, seems to belong to reflex actions. It is well known that an irritation of the skin and of the mucous membranes may easily produce certain reflex movements, which very rarely take place after an irritation of the trunks of the sensitive nerves. For instance, coughing is almost a constant result of an irritation of the mucous membrane of the larynx and of the bronchial tubes, while it is very rarely produced by an irritation of the trunk of the par vagum. Something similar exists for the production of convulsive fits when the face is irritated in animals upon which the spinal cord has been injured. If we lay bare the nerves of the face and neck of these animals, we find that even the greatest irritations upon them do not produce a fit. Besides, if we dissect a large piece of the skin of the face, so as to let it be in connection with the nervous centres only by the suborbitory nerve, we find that the irritation of this piece of skin is still able to produce convulsions, while the irritation of the very nerve which connects it with the brain does not produce any. It seems, therefore, that it is in the cutaneous ramifications of certain nerves of the face and neck that resides the faculty of producing convulsions in the animals upon which I have injured the spinal cord. There is, in that case, as I will show hereafter, something resembling what takes place in man in cases where a ligature around a limb is sufficient to prevent a fit of epilepsy.

§ V. What is the nature of the fits that we find in animals upon which the spinal cord has been injured? I think these fits ought to be considered as epileptic. The following description of these convulsions will show that, if they are not positively epileptic, they are at least epileptiform. When the attack begins, the head is drawn first, and sometimes violently, towards the shoulder, by the contraction of the muscles of the neck, on the side of the irritation; the mouth is drawn open by the contraction of the muscles of the neck, which are inserted upon the lower jaw, and the muscles of the face and eye (particularly the orbicularis) contract violently. All these contractions usually occur simultaneously. Frequently at the same time, or very nearly so, the animal suddenly cries with a peculiar hoarse voice, as if the passage of air were not free through the vocal cords, spasmodically contracted. Then the animal falls, sometimes on the irritated side, sometimes on the other, and then, all the muscles of the trunk and limbs that are not paralyzed become the seat of convulsions, alternately clonic and tonic. The head is alternately drawn upon one or the other side. All the muscles of the neck, eyes and tongue contract alternately. In the limbs, when the convulsions are clonic, there are alternative contractions in the flexor and the extensor muscles. Respiration takes place irregularly, on account of the convulsions of the respiratory muscles. Almost always there is an expulsion of fæcal matters, and often of urine. Sometimes there is erection of the penis, and even ejaculation of semen.

These are the features which render these fits very much like epilepsy. But they seem to differ from this disease, by the three following characters: 1st. The animals sometimes cry during the fits, when they are irritated, and it seems, therefore, that they have not lost their sensibility. Now as the loss of sensibility is considered a symptom essential to epilepsy, it appears that we ought not to consider as epileptic the convulsions existing in these animals. But, we cannot admit this as a decisive objection, when we remark that frequently they seem to be deprived of sensibility, and that, in man, during true fits of epilepsy, there are sometimes periods where sensibility is not lost. 2d. These animals usually have no foam at the mouth, and this symptom has been considered by many writers as essential to epilepsy; but there can be no doubt that there are cases of epilepsy without any foam. Besides, we may easily understand why there is no foam ordinarily in animals: usually their fits do not last long enough. 3d. The fits in these animals are most frequently a series of fits lasting two or three minutes, and separated one from the other by a period of one or two minutes, during which the animals are able to rise and to stand on their feet. In this respect these animals differ from the majority of epileptic men, who have not a recurrence of fits after so short a period of calm; but there are cases of rapidly-recurring fits in man, and therefore we cannot deny that the fits of these animals are true epileptic fits, on the ground that they have that peculiar character of rapid recurrence.

The apparent differences between the fits in animals which have had the spinal cord injured, and true epilepsy in man, ought not, therefore, to prevent our considering them as epileptic fits. Not only the convulsions resemble those of true epilepsy, but the fits are not mere accidents, and they come by series of two or three, once a week, once a day, or even ten or twenty times a day, and the disease lasts for years. Besides, we find, after long and violent fits, that these animals are, for a time, in a state of drowsiness, like men after epileptic convulsions. It seems rational to conclude, from this discussion, that if the convulsions of these animals are not truly epileptic, they are at least epileptiform.

§ VI. The facts expressed in the preceding parts of this paper lead to many interesting conclusions. *First*, they give a positive proof that an injury to the spinal cord may be the cause of an epileptiform affection. *Secondly*, they show a wonderful relation between certain parts of the spinal cord and certain branches of some of the nerves of the face and neck. *Thirdly*, they show that epileptiform convulsions may be the constant consequence of slight irritations upon certain nerves. *Fourthly*, they show that even when an epileptiform affection has its primitive cause in the nervous centres, some cutaneous ramifications of nerves, not directly connected with the injured parts of these centres, have a power of producing convulsions, that other nerves, even directly connected with them, have not. *Fifthly*, they show that the cutaneous ramifications of certain nerves may have the power of producing convulsions, while the trunks of these nerves have not this power.

§ VII. The constant appearance of a disease very much resembling epilepsy, after certain injuries to the spinal cord, in animals, will perhaps settle the undecided question whether epilepsy, in man, may originate from an alteration of the spinal cord or not. It seems very strange that physicians have been so unwilling to admit that the spinal cord could be the seat of the primitive cause of epilepsy, when they admit that any nerve or any part of the encephalon, being altered, may produce epilepsy. The seat of this disease seems to be together in the part of the brain where resides the faculty of Perception and of Volition, and in the part of the cerebro-spinal axis endowed with the reflex faculty; but, whatever may be thought on this subject, it seems quite certain, from facts observed in man and in animals, that epilepsy may be produced by various kinds of alterations of the encephalon, of the spinal cord and of a great many nerves. In other words, the peculiar disturbance of the cerebro-spinal axis which constitutes epilepsy, may be generated by alterations of various parts of this nervous axis and by many nerves. This view does not agree with that of the most distinguished among the recent writers upon epilepsy. They have hardly spoken of the influence of the alterations of the spinal cord upon the production of epilepsy. For instance, M. Delasiauve (*Traité de l' Epilepsie*, 1854, pp. 174–181) does not speak at all of this influence, and we find that he places a case of epilepsy with an hypertrophy of the spinal cord among many other cases forming a series of doubtful or equivocal alterations. Hasse does not pay more attention than Delasiauve to the share of the spinal cord in the causation of epilepsy. He seems to take notice only of the influence of the alterations of the encephalon. (*Krankheiten des Nervenapparates*, 1855, pp. 266–67.) Romberg (*Lehrbuch der Nervenkrakheiten des Menschen*, 3d edition, 1855, vol. i. part 2, p. 686) has written only a few lines on the relations between alterations of the spinal cord and epilepsy. He thinks that some of the facts related by Oliver d'Angers prove the existence of these relations.

M. Bouchet, who had, in a paper with M. Cazanvielh (*Archives de Medec., etc.*, 1825, t. ix.), mentioned some cases of diseases of the spinal cord with epilepsy, has tried to show in a recent paper, (*Annales Médico-Psychol.*, 1853) that epilepsy is usually connected with the hippocampus major (*cornn ammonis*).

If we take notice of this fact that the spinal cord is very rarely examined, we understand that although the number of cases on record, as far as I know, of alterations of this organ in epilepsy, amounts only to about fifty, there is an immense number of cases in which after death from the so-called idiopathic tetanus, the brain was examined, but not the spinal cord. In these cases, particularly where nothing is found in the brain able to account for the disease, it should have been of the greatest importance to examine the spinal cord. Such a neglect is a great fault, particularly since the publication made by Esquirol on the result of his autopsies. In the corpses of ten epileptics, Esquirol (*Traité des Maladies Mentales*, 1838, vol. i., p. 311) found nine times, various alterations of the spinal cord or of its membranes. In four cases, the spinal cord was softened, particularly in the lumbar region; nine times there were lenticular concretions in the arachnoid, some of which were cartilaginous, some osseus; once there were a great many hydatids in the cavity of the arachnoid.

Mitivie, quoted by Esquirol (*loc. cit.*, p. 311), found concretions in the arachnoid in two children who died from epilepsy.

Two cases of chronic meningitis with epilepsy, have been recorded by M. Clot. (*Rech. and Observ. sur le Spinilis*, 1820.) One case of this kind is related by Ollivier d'Angers (*Traité des Maladies de la Moelle épinière*, 3ème *edit.*, 1837, vol. ii., p. 319).

Calmeil (*De l'ëpil. sous le rapport de son siège*, 1824) speaks of four epileptics, in two of whom the spinal arachnoid contained many cartilaginous plates, while in the two others the density of the spinal cord was considerably increased.

Bouchet and Cazanviehl have found, in many cases, circumscribed softenings of the spinal cord, and other alterations of this organ and its sheath.

Forget, quoted by Ollivier d'Angers (*loc. cit.*, vol. ii., p. 571), has seen two very important cases, which have a great analogy with what I have found in animals.

Jendrin, quoted by Ollivier (vol. ii., pp. 502 and 520), has found in two epileptics a tubercle in the cervical region of the spinal cord.

Barthez and Rilliet (*Traité des Maladies des Enfauts*, 2d edit., 1854, vol. iii., p. 589) relate a very curious case in which epilepsy existed in a girl, who had an angular curvature of the spine in the dorsal region. The symptoms were very much the same as those existing in my animals, and, as it is in them, there was no foam at the mouth. There was no alteration in the nervous centres, except in the dorsal region of the spinal cord, which was almost liquefied. This softening occupied the whole of the cord transversely, and was about one centimetre long.

I might add many other cases of alteration of the spinal cord in epileptics, recorded by writers of the previous centuries, such as Bouet, Lieutaud, Morgagni, Musel, &c. In the work of Portal (*Observ. sur la Nat. et le Traitement de l' Epil.*, 1827, p. 28) there is a curious case of epilepsy with a dilatation of the central canal of the spinal cord, which was filled with water.

If epilepsy has truly been the result of an alteration of the spinal cord in all or some of the above cases, it might be asked why there are so many cases of diseases or injuries of the spinal cord without epilepsy. This objection loses its value when we remark that every day there are cases of tumors and various alterations of the brain without epilepsy, and that, nevertheless, no one doubts that this disease is sometimes produced by such lesions. Besides, I have found that certain kinds of injury to the spinal cord, in animals, produce much more frequently than others an epileptiform affection, and there is only one kind of injury which seems to produce it constantly. This injury consists in a section of the whole of a lateral half of the spinal cord. I do not kuow of a single case, in man, where life has been saved after such an injury had been done to the spinal cord. In some cases, where, probably, a great part of the lateral half of this organ had been divided transversely, there has been no epilepsy. Such a case is recorded by Morgagni (*De sed. & causis morborum*, ep. 53, § 23); another by Boyer (*Traité des Maladies Chirurg.*, 1ère edit., vol. vii., p. 9), and a third by my friend, M. Vignès (*Moniteur des Hôpitaux*, 1855, p. 838). In animals, after an incomplete transversal section of a lateral half of the spinal cord, epilepsy is not very frequently produced. Therefore the negative facts concerning the influence of this injury in man, cannot be considered as a proof that man does not resemble animals in this respect.

I think the following conclusions may be drawn from all that I have said concerning the influence of alterations of the spinal cord upon the production of epilepsy: 1st. There cannot be any doubt that in animals certain injuries to the spinal cord frequently produce an epileptiform affection, if not true epilepsy. 2d. That in man there are a great many cases which seem to prove that alterations of the spinal cord may cause epilepsy.

Now, as we well know that the spinal cord has the same organization and the same vital properties, in animals and in man, it seems, from the first of these conclusions, that it may be stated more positively than I have done in the second, that epilepsy may result from alterations of this nervous centre.

§ VIII. Physicians admit now, two kinds of epilepsy, one of centric and the other of peripheric origin. I will try to show that although it seems to be of peripheric origin, it may, in some cases, be in reality of centric origin.

In animals, after an injury of the spinal cord, if we did not know that this injury exists and is the first cause of the disease, we should be led to admit that it is of peripheric origin, in finding that an irritation upon a very limited part of the spine produces fits. In a very important case of epilepsy recorded by Odier, the same thing has existed as in my animals. For many years the disease seemed to be of peripheric origin, and the autopsy has revealed that this was a mistake. This case is so interesting, in many respects, that I will give here a summary of its principal points.

Case I.—A man had frequent *cramps* in the little finger of the right hand. The contractions went on increasing in extent and frequency; they by degrees extended to the fore-arm, the arm and the shoulder, always beginning in the little finger. At last they arrived at the head, and true fits of epilepsy, with loss of consciousness, took place. By means of two peculiar ligatures round the arm and the forearm, and which the man could tie easily, when he felt the first contractions of the little finger, the attacks were prevented at every threatening for two or three years. Unfortunately, he eat and drank too much, and, being intoxicated, he forgot the ligature when the initial cramp appeared, and then he had a violent fit. From this time the ligature had no more influence over the fits; they became very frequent and always began in the little finger. Paralysis came on, and the patient died in coma. *Autopsy.*—An enormous tumor was found in the left side of the brain, below a place where the cranium had been wounded long before. (*Odier, Manuel de Médecine Pratique*, 2de edit., 1811, p. 180).

This case, and the facts observed in man, positively show that the apparent outside origin of epileptic fits does not prove that there is not an organic cause in the nervous centres.

